# Driver anomaly detection in cargo terminal

**DOI:** 10.1016/j.heliyon.2024.e41567

**Published:** 2024-12-31

**Authors:** Shahab Emaani, Abbas Saghaei

**Affiliations:** a Master's degree, Department of Industrial Engineering, Technical and Engineering Faculty, Research Science Unit, Islamic Azad University, Tehran, Iran; b Professor, Department of Industrial Engineering, Technical and Engineering Faculty, Research Science Unit, Islamic Azad University, Tehran, Iran

**Keywords:** Anomaly detection, Fraud detection, Cargo, Terminal, Machine learning

## Abstract

The Iranian road transportation sector, comprising about 500,000 owner-operator drivers, faces rising syndication challenges, leading to disruptions and driver refusals in some provinces. Drivers highlight the urgent need for load distribution improvements within terminals. This study investigates anomaly detection by drivers in cargo terminals, starting with the evaluation of driver assumptions through K-means clustering. The study confirms drivers' assertions regarding those who handle more cargo in less waiting time. Subsequently, Isolation Forest, KNN, and HBOS algorithms are applied to detect abnormal behavior using data mining techniques. Results reveal three distinct driver groups, with a notable proportion (98 %) of anomalies concentrated in one group. This study sheds light on the critical syndication issue of anomaly detection in cargo terminals by drivers, offering valuable insights for researchers and shipping practitioners. Moreover, limited research on theft prevention renders conventional methods ineffective, highlighting the overlooked use of clustering in prior literature reviews focused on case study analysis.

## 1 Introduction

Efficient and secure operations within cargo terminals are critical components of global logistics, directly impacting economic stability and the effectiveness of supply chains. Anomaly detection, a foundational aspect of contemporary data analytics, is instrumental in safeguarding operational integrity. This study examines the Iranian road transportation sector, which accounted for 10.8 % of Iran's GDP in 2018 and includes approximately 500,000 owner-operator drivers. These drivers, while essential to the national economy, encounter systemic challenges such as inequitable load distribution and syndication-related disruptions, which have led to operational inefficiencies in several key provincesFig. 2, Fig. 3, Fig. 4, Fig. 5, Fig. 6.

This research highlights the significance of anomaly detection in the context of cargo terminals, emphasizing its capacity to identify fraudulent activities, enhance operational safety, and improve efficiency. As a specialized domain of data analysis, anomaly detection enables the identification of patterns and deviations from expected norms [1]. Cargo terminals, as critical nodes within supply chains, present unique operational challenges. Detecting driver anomalies requires the identification of behavioral deviations that may signal inefficiencies or fraudulent practices. Insights derived from such analysis mitigate operational risks while fostering cost-efficiency and bolstering stakeholder confidence.

We utilize advanced clustering methods, such as K-means, alongside anomaly detection algorithms like Isolation Forest, HBOS, and KNN. These methodologies are validated through their application to real-world datasets from Iranian cargo terminals, demonstrating their effectiveness in distinguishing patterns that indicate anomalies. Furthermore, the results highlight actionable insights for addressing systemic inefficiencies and enhancing terminal operations.

By bridging the gap between theoretical methods and practical applications, this study contributes to the broader discourse on anomaly detection's role in logistics. The findings serve as a basis for future research and actionable strategies in anomaly detection, particularly in understudied contexts such as cargo terminals.

The subsequent sections of this paper are structured as follows: Section 2 provides an overview of the extant literature pertaining to driver anomalies in terminals. Section 3 introduces the data-driven methodology, while Section 4 delineates the empirical study conducted at a terminal in Iran. Section 5 presents the results derived from the application of the methodology and experimental conclusions. Lastly, Section 6 deliberates on the paper and outlines future perspectives.

## 2 Literature review

The increase in the number of frauds in various fields has become a serious challenge in the last decade [2]. Considering the advancement of theft technology, illegal activities have increased significantly [3]. Due to the lack of effective systems to predict fraud, many frauds are detected after they occur [4]. Considering the importance of the ports and the financial value of the cargoes that pass through them, securing the ports is a significant necessity [5]. Most port and terminal risk management focuses on the development, organization, and commercial issues. Strategic collaborations in order to reduce risk in the transportation network has been considered since the past [6]. However, few articles have tried to reduce the risk of sending cargo using quantitative and qualitative methods. Regarding the presented research, most of the articles have focused on avoiding theft by means of software methods, hardware facilities and managerial measures [7]. Increasing urban population, reducing costs and increasing international trade can be considered as the main results of creating security in ports and terminals.

Among the limited researches on cargo theft in the current literature, most of them have focused on countermeasures against various types of cargo theft accidents and other research related to discovering the nature of cargo theft [8]. Few articles have analyzed the factors affecting cargo theft in a categorized way [9,10] used machine learning methods to predict the probability of cargo theft in railway transport. The effectiveness of various methods in predicting cargo theft was evaluated in this article. Ref. [11] studied intelligent fraud detection systems using a Bayesian network that predicts the presence of goods on the cargo list of shipments. It can be understood from the obtained results that intelligent fraud detection systems performed better in detection of miscoding compared to random audits. A prediction model was presented in order to reduce the risk of cargo theft [12]. Real-time tracking and control measures are used to reduce the risk of goods theft in this article.

A review article on fraud detection by machine learning methods was presented. In this article, the role of artificial intelligence in the field of automatic prediction of fraud patterns is challenged [13]. A comprehensive analysis of various financial fraud techniques was presented [14]. The focus of this article was on credit card fraud using machine learning methods. A data-driven approach is used to predict the theft risk of bulk cargo in ports based [7]. In this article, the performance of different binary classifiers was compared and based on that, the best algorithm was [15] used big data analytics to identify an unusual pattern to detect and prevent fraud in various sectors. Different predictive analytics options are used in this paper to handle massive data and their pattern. A data-driven approach is developed to formulate predictive models for bulk cargo theft in port [16]. The results of the Bayesian network were analyzed in a real case study.

Due to the movement of many means of transportation to the terminals and the existence of valuable cargo, they can be considered as one of the main centers for cargo thieves [17]. Considering that a major part of the planning and logistics for transportation is done in the terminals, it seems necessary to plan to establish security in the terminals [18]. Iran's road transport industry associations have encountered several issues in recent years, resulting in drivers refusing to transport cargo in several provinces, negatively impacting the economy and many businesses. Due to technological advancements and an increase in data volume, traditional fraud detection methods, such as survey-based techniques, are no longer optimal for bold prediction. High-priced sample inspections are currently the most prevalent and effective method for combating fraud. Due to little research that has been done in the field of theft prevention, they are not effective enough for preventing theft practically. In Table 1, a comparison of the contributions of the past articles with the current article is made.

**Table 1:** A comparison of the contributions of previous articles with this article.

Article	Data-Driven	Scope	Clustering	Case Study
Wu et al. [9]		Logistics Systems		
Triepels et al. [11]		International Shipping		
Jha et al. [15]		Port		
Lorenc et al. [10]		Railway Transport		
Liang et al. [8]		Freight Supply Chain		
This Article		Terminals		

In recent years, various studies have explored the combination of K-means clustering and Isolation Forest (IF) for anomaly detection in different domains. For instance, the work by Ref. [19] “K-means-based Isolation Forest” utilized the integration of K-means and IF for detecting anomalies in transportation data. This approach aligns closely with the methodology employed in this study, where K-means clustering is used to group drivers based on operational characteristics, and Isolation Forest is applied to identify outliers that deviate from expected behavior.

While the study by (Karczmarek) focuses on transportation data, our research extends this approach to the cargo terminal environment, where operational dynamics, such as load handling and waiting times, differ significantly. By applying the same principles of combining K-means clustering with IF, we aim to uncover inefficiencies and potential fraudulent behavior within cargo terminal operations. The similarities between the two studies highlight the robustness of using K-means and IF for anomaly detection, regardless of the specific context. However, our study introduces novel insights into the operational challenges faced by cargo terminals, including the impact of geographical location and load allocation practices on driver behavior.

The use of these methods in both studies underscores their versatility and effectiveness in identifying patterns of behavior that deviate from the norm, providing valuable insights that can inform operational improvements in various sectors, including transportation and logistics.

According to the studied articles and the gap obtained from the comparative table of past researches, an article has been presented in order to detect driver anomalies in terminals using clustering methods. Some of the main contributions of this article are as follows:

• Clustering of drivers using K-means method
• Categorizing the types of anomalies
• Controlling the accuracy of clustering by silhouette coefficient technique
• Anomalies detection using KNN, HBOS and Isolation Forest algorithms

## 3 Methodology

In this study, our approach to fraud detection involves a systematic breakdown into distinct phases. Following a survey involving approximately 400 drivers, a notable observation emerged: certain individuals within each terminal exhibit patterns suggestive of potential fraudulent behavior. Specifically, these individuals not only handle more cargo than their counterparts but also demonstrate significantly lower average time per task. To validate this observation, we initially endeavored to cluster the drivers utilizing the K-means algorithm, aiming to discern any underlying patterns. However, the effectiveness of these clusters warrants evaluation through the silhouette score metric to ensure robustness. Should this assumption prove substantiated, our subsequent focus will delve deeper into the detection of anomalous drivers, employing various anomaly detection methodologies.

Before embarking on the utilization of algorithms, it is imperative to first establish the categories of anomaly detection. Broadly speaking, there exist three principal types of anomalies that are typically encountered in datasets. These categories serve as fundamental frameworks for anomaly detection methodologies.

• Point anomaly: is the most widely employed in scientific research.
• Contextual anomaly: also known as a conditional anomaly, is an anomalous occurrence in a particular context. Every instance is made up of contextual and behavioural attributes. The values of behavioural attributes indicate the context-specific anomaly of an instance. Typically, contextual anomalies occur in time series data or spatial data. Collective anomaly refers to a group of related data instances that are out of the ordinary for the entire data set. In contrast, each data instance may not be anomalies in other circumstances.
• Collective anomalies are most prevalent in sequence, graphical, and spatial data. A point anomaly detection problem or a collective anomaly detection problem could be transformed into a contextual anomaly detection problem when a context is considered.

Outlier detection stands as one of the foremost techniques utilized for detecting fraud, particularly in low-dimensional datasets. An outlier, in essence, represents an observation that diverges to such a notable extent from the rest of the dataset that it prompts speculation regarding its origin, potentially suggesting it was generated by a distinct mechanism or process. This method serves as a vital tool in identifying irregularities or fraudulent activities within data, offering valuable insights into anomalous behavior that might otherwise go unnoticed [20]. Anomalous behavior is becoming increasingly significant across a wide array of applications. However, detecting anomalies in streaming data poses significant challenges owing to the inherent characteristics of such data streams. These challenges include the rapid generation of data, the perpetual nature of data flow, vast volumes of data, and the occurrence of concept drift over time. The process of identifying abnormal behavior or detecting outliers involves pinpointing data patterns that deviate from the expected patterns exhibited by other data. To address this task, a multitude of methods for detecting outliers exist, typically falling into four distinct categories [21].

• Distance-based
• Density-based
• Modal-based
• Isolation-based

Within each of these categories lie significant algorithms, each designed to address specific aspects of anomaly detection. Among these, the K-nearest neighbors (KNN) algorithm stands as a notable example within the distance-based methods. KNN operates by assessing each data point in relation to its nearest neighbors, determining its proximity within the dataset. Points that exhibit considerable distance from their nearest neighbors are classified as outliers, representing instances that deviate significantly from the norm within the dataset [22].

Another model under consideration for anomaly detection is Histogram-based Outlier Score (HBOS), which falls under the category of density-based methods. HBOS operates under the assumption of feature independence and evaluates the degree of anomalies by constructing histograms for each feature in the dataset. In the context of multivariate anomaly detection, HBOS computes a histogram for each individual feature, assigning a score to each feature independently. These scores are subsequently combined to generate an overall anomaly score, providing a comprehensive assessment of anomalous behavior within the dataset [23]. Through our experimental analyses, it was observed that HBOS exhibits a remarkable speed advantage, being up to five times faster than density-based algorithms and up to seven times faster than nearest-neighbor methods. This notable performance enhancement positions HBOS as a promising anomaly detection solution, particularly in scenarios where computational efficiency is paramount [24].

Isolation Forest emerges as another pivotal algorithm in the realm of anomaly detection. This algorithm, akin to a random forest, operates on the principles of decision trees. It begins by randomly selecting a variable and subsequently isolating data points based on this selection. However, it's crucial that the chosen data points fall within the minimum and maximum values of the attribute. Unlike traditional methods reliant on distance or density measurements, Isolation Forest discerns anomalies through a distinctive approach, which significantly reduces the computational overhead associated with distance-based and density-based techniques. Moreover, Isolation Forest boasts linear time complexity and memory requirements, rendering it highly scalable for handling extensive datasets and high-dimensional problems featuring numerous extraneous attributes [25].

Outliers were detected using three distinct anomaly detection algorithms: HBOS, iForest, and KNN. Each method identified outliers based on different principles, providing complementary perspectives on the dataset. To enhance reliability, only anomalies detected by at least two methods were retained for further analysis. This consensus approach reduced false positives and ensured robust anomaly detection across clusters.

Cross-validation was not utilized in this study as the primary focus was on anomaly detection rather than predictive modeling. Given that anomaly detection is an unsupervised learning task, the application of cross-validation—which is predominantly employed in supervised learning to assess the generalizability of predictive models—was deemed unnecessary and not relevant to the objectives of this research.

### 3.1 Feature selection

Feature selection plays a crucial role in anomaly detection, as the chosen variables must accurately capture the underlying operational dynamics and behavioral patterns within the dataset. The features selected for this study were carefully chosen to represent key aspects of driver behavior and terminal operations, enabling effective clustering and anomaly detection. These features are as follows:

• Local: This binary feature indicates whether a driver is local (1) or non-local (0). Local drivers may exhibit distinct patterns, such as shorter waiting times or improved access to facilities, making this feature vital for distinguishing between normal and abnormal behaviors. Since K-means clustering relies on numerical data and Euclidean distance calculations, the nominal Local feature was encoded using binary encoding. This approach preserved the geographical distinction while ensuring compatibility with the clustering algorithm, enabling the analysis to incorporate regional differences in driver behavior.
• Ticket Count: Represents the number of turn tickets a driver receives. High ticket counts may signal unusual or potentially fraudulent activity, particularly when disproportionate to other metrics such as waiting time or loads handled. This feature helps identify drivers whose activity may warrant further investigation.
• Waiting Time (days): Measures the total time a driver spends waiting in the terminal. Deviations from typical waiting times—whether significantly long or short—can highlight inefficiencies, operational delays, or irregular practices that may indicate anomalies.
• Average Waiting Time (days): This feature normalizes the waiting time per load, providing a clear measure of operational efficiency and fairness in load distribution. Significant deviations from average waiting times can indicate systemic issues or anomalies in the terminal's operations.
• Count Loads: Reflects the total number of loads handled by a driver. Anomalous load counts, whether unusually high or low, may suggest preferential treatment, manipulation of the system, or issues related to load distribution (see Table 2).

**Table 2:** Relevant elements of fraud detection.

Feature	Explanation	Scale
Local	0: not local	–
	1: local	
Ticket count	Continuous variable	1 to 124
Waiting time (days)	Continuous variable	0 to 74
Average waiting time (days)	Continuous variable	0 to 34
Count loads	Continuous variable	0 to 123

These features were selected due to their direct relevance to addressing key challenges in terminal operations, such as fraud detection, efficiency optimization, and equitable load distribution. Their inclusion allowed for a comprehensive analysis of driver behavior within the dataset.

In addition to these features, the dataset contained temporal information related to driver activities over time. To facilitate compatibility with clustering and anomaly detection methods, raw time data was aggregated into features like Waiting Time (days) and Average Waiting Time (days). This transformation normalized the unequal time intervals within the dataset and enabled meaningful comparisons between drivers, focusing on operationally relevant metrics.

The dataset underwent extensive preprocessing to ensure the features were properly prepared for analysis. Nominal features, such as Local, were binary-encoded, while numerical features, including Waiting Time and Count Loads, were normalized using min-max scaling to ensure consistency in scale. Additionally, aggregated metrics like Average Waiting Time were computed to standardize time-related data across drivers. These preprocessing steps ensured the compatibility of the dataset with the clustering and anomaly detection algorithms, while maintaining the integrity and relevance of the features for analysis.

### 3.2 Empirical study

Out of Iran's 53 cargo terminals, only 23 include a hall facility. Nonetheless, for this study, four terminals were specifically chosen based on expert recommendations and factors such as activity volume, importance, and diversity of goods handled. Subsequently, all databases were meticulously inspected by visiting the selected terminals and scrutinizing the data. However, only three out of the six databases were deemed relevant for the study's objectives: parking, queuing system, and freight announcement center. Each of these databases contained approximately 12 features and 50,000 rows of data. Following extensive preprocessing and data cleaning, the dataset was streamlined to encompass 22 features and 350,000 rows. The temporal scope of the data spans from January 2019 through March 2019.

## 4 Results

To maximize the utility of the data, an initial step involved identifying 75,000 drivers. Subsequently, a diverse set of features was employed to extract relevant information crucial for fraud detection purposes. Prior to applying the K-means algorithm, the data underwent a scaling process to ensure uniformity and effectiveness in feature extraction.

**Fig. 1:**
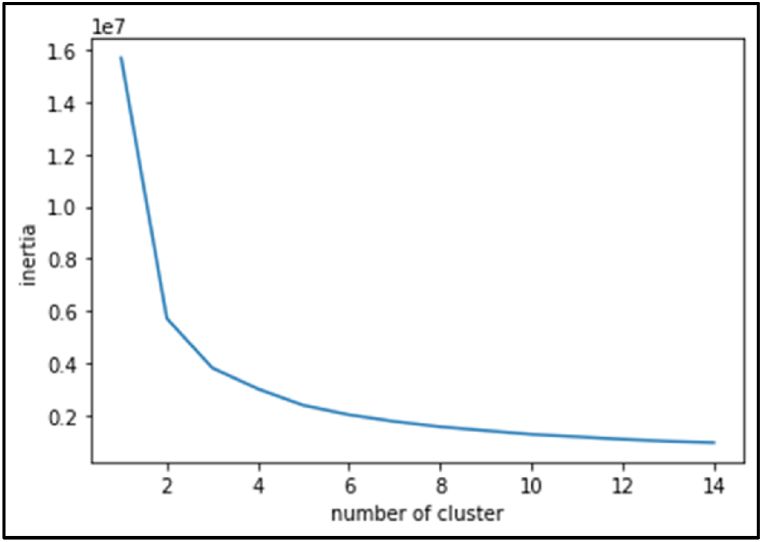
Number of clusters.

Subsequent to the meticulous preparation of the dataset, the K-means algorithm was deployed to discern the ideal number of clusters for categorizing the drivers. This analysis spanned across a spectrum of clusters, ranging from 1 to 15, facilitating a comprehensive exploration of the dataset's underlying structure and enabling the identification of the most appropriate clustering configuration (see Fig. 1).

To determine the optimal number of clusters, the elbow method was employed. This heuristic technique, commonly utilized in cluster analysis, involves plotting the explained variation against the number of clusters. Subsequently, the point on the curve where a noticeable bend or “elbow” occurs is selected as the optimal number of clusters to utilize in the analysis [26]. As a result, three clusters were chosen based on the analysis. The findings derived from the clustering process are delineated in Table 3.

**Table 3:** Results of K-means clustering.

Cluster	The number of drivers	Local drivers %	Average turn ticket	Average wait time for each load (day)	Average number of loads
Group 0	15678	16.65 %	3	11	2
Group 1	16302	54.64 %	10	3	8
Group 2	43335	28.71 %	2	1	1

Group 0, represented by the color blue, comprises individuals characterized by prolonged waiting times in queues for their intended loads. On average, members of this group spend approximately 11 days waiting in line.

Group 1, depicted in red, consists of individuals who handle a substantial number of loads but experience considerably shorter waiting times compared to Group 0. Members of this group typically wait for only two days per load. Approximately 54 % of the local population falls within this category.

**Fig. 2:**
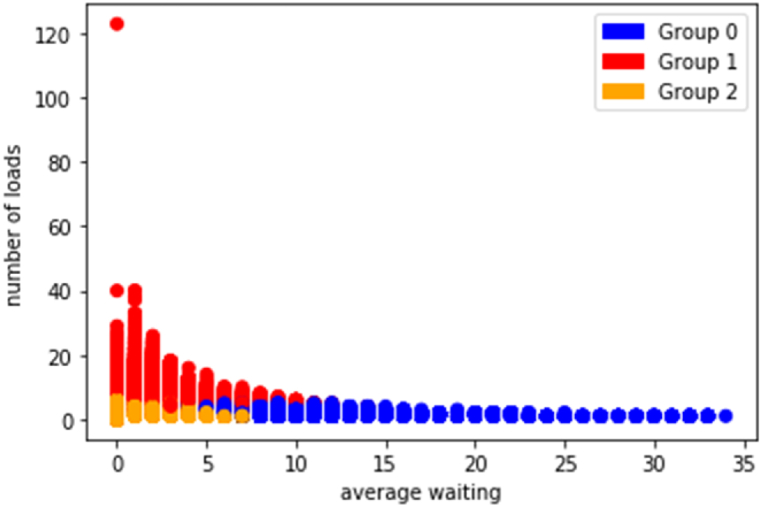
Clusters of drivers.

Group 2, depicted in orange, encompasses individuals characterized as average in their load handling and waiting times. Members of this group typically undertake an average of two loads and endure a waiting period of one day per load (see Fig. 2).

**Fig. 3:**
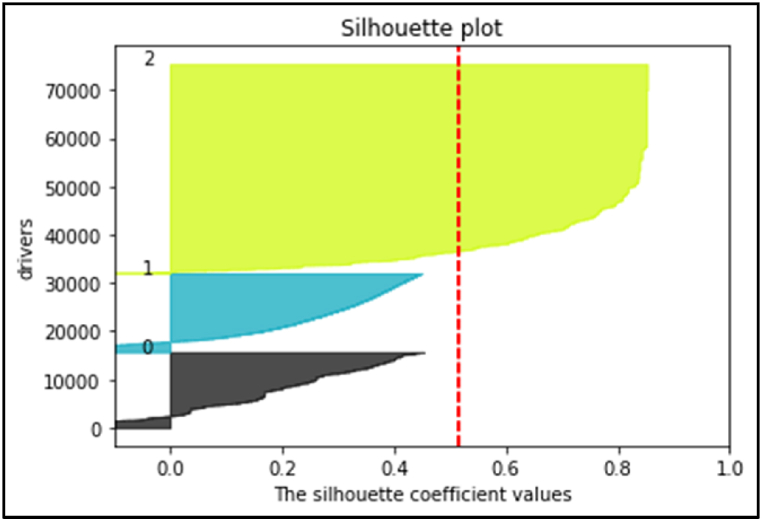
Silhouette values.

To assess the quality of data clustering, we employ the silhouette coefficient, a technique utilized for interpreting and validating the consistency of data clusters (see Fig. 3). This technique furnishes a concise graphical representation of the clustering quality for each object within the dataset. The silhouette value quantifies how closely an object resembles its own cluster (cohesion) in contrast to other clusters (separation). Ranging between 1 and +1, a high silhouette value indicates that the object is well-suited to its own cluster but poorly matched to neighboring clusters. A clustering configuration is deemed suitable if the majority of objects exhibit high silhouette values. Conversely, if numerous points yield negative or low silhouette values, it suggests that the clustering configuration may contain either an excessive or insufficient number of clusters [27].

(1) $$a\left(i\right)=\frac{1}{\left|c_{i}\right|-1}\sum_{j\in c_{i},i\neq j}d\left(i,j\right)$$

(2) $$b\left(i\right)=\min_{k\neq i}\frac{1}{\left|c_{k}\right|}\sum_{j\in c_{k}}d\left(i,j\right)$$

(3) $$s\left(i\right)=\frac{b\left(i\right)-a\left(i\right)}{\max\left\{a\left(i\right),b\left(i\right)\right\}},if\left|c_{i}>1\right|$$

$i\in c_{i}$: data point i in cluster $C_{I}$, d(i,j): distance between data points i and j in cluster $C_{I}$, Our mean silhouette score is 0.56. Thus, we can conclude that our clustering is appropriate for our dataset.

Following the clustering of drivers, it became evident that a notable proportion exhibited behaviors distinct from those observed in other groups, confirming the validity of our initial assumption regarding driver behavior. Subsequently, our focus shifted towards a more detailed examination of the abnormal behaviors displayed by these drivers. To address this, we adopted three distinct analytical approaches aimed at comprehensively assessing and understanding the nature of these anomalies.

**Fig. 4:**
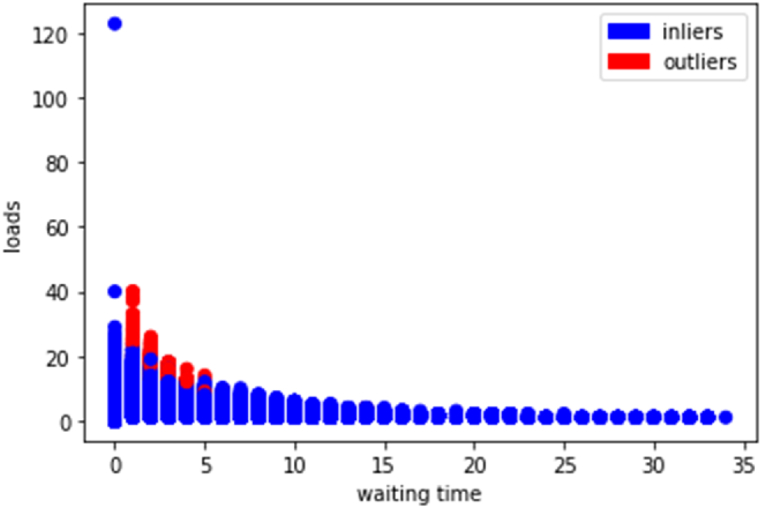
HBOS results.

HBOS (Histogram-based Outlier Score) operates by calculating individual histograms for each dimension d within the dataset. These histograms are constructed irrespective of the data type, whether categorical, fixed-width, or dynamic-width, where the height of each bin reflects an estimation of the density. Subsequently, the HBOS of each instance p is determined by assessing the heights of the bins in which the instance falls. This process enables the identification of outliers based on their deviation from the expected density patterns within the dataset (see Fig. 4).

(4) $$HBOS\left(p\right)=\sum_{i=0}^{d}\log\;\left(\frac{1}{{hist}_{i}\left(p\right)}\right)$$

The score is computed by multiplying the inverses of the estimated densities under the assumption of feature independence. Utilizing this approach, HBOS successfully identified 688 data points as outliers within the dataset.

**Fig. 5:**
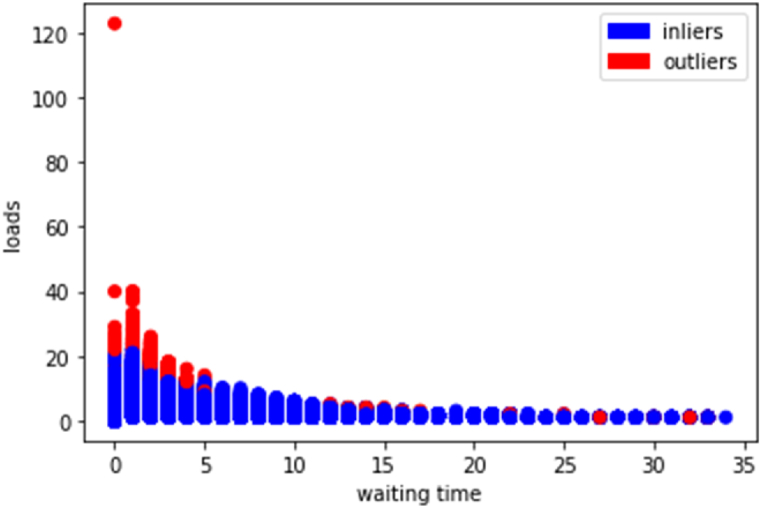
IForest results.

In Isolation Forest, the algorithm randomly selects a subset of features and data points from the dataset. It then constructs a decision tree structure, where each branch represents a binary split based on a randomly chosen feature and threshold value. This process continues recursively until each data point is isolated in its own leaf node or a predefined maximum depth is reached. By repeatedly partitioning the data in this manner, anomalies are expected to be isolated in fewer partitions, requiring fewer splits to separate them from the majority of normal data points. The scores assigned to data points are based on the depth of the leaf node in which they reside. Anomalies typically end up in shorter paths within the tree, resulting in lower scores compared to normal data points. Therefore, lower scores indicate a higher likelihood of being an anomaly [25]. The Isolation Forest (iForest) algorithm successfully identified approximately 754 outliers within the dataset. These outliers were detected based on their characteristic placement within the decision tree structure, where anomalies typically reside in shorter paths, resulting in lower scores compared to normal data points (see Fig. 5).

(5) $$c\left(m\right)=\left\{ \begin{array}{c} 2H\left(m-1\right)-\frac{2\left(m-1\right)}{n}\;for\;m>2\\ 1\;for\;m=2\\ 0\;otherwise \end{array}\right.$$

(6) H(i)=ln(i)+0.5772

(7) $$s\left(x,m\right)=2^{\frac{-E\left(h\left(z\right)\right)}{c\left(m\right)}}$$

n: testing data, m: sample size, H: harmonic number, C(m): average of h(x), E(h(x)): is the average value of h(x) from a collection of iTrees

The K-nearest neighbors (KNN) method is a commonly employed approach for anomaly detection. It works by calculating the distances between each data point and its nearest neighbors in the dataset. The formula for the K-nearest neighbors (KNN) algorithm involves calculating the distance between a target data point x and its nearest neighbors in the feature space. The distance metric commonly used is Euclidean distance, but other distance metrics such as Manhattan distance or cosine similarity can also be employed depending on the characteristics of the data.

For a given target data point x, the distance $d\left(x,x_{i}\right)$ between x and each of its neighbors ${\;x}_{i}$ is computed. Then, the K nearest neighbors are identified based on the smallest distances. The formula to calculate the Euclidean distance between two data points x and $x_{i}$ in n dimensions is:

(8) $$d\left(x,x_{i}\right)=\sqrt{\sum_{j=1}^{n}{\left(x_{ij}-x_{i}\right)}^{2}}$$

Where: $x_{ij\;}$ represents the j-th feature of the i-th neighbor. $x_{i}$ represents the j-th feature of the target data point x.

**Fig. 6:**
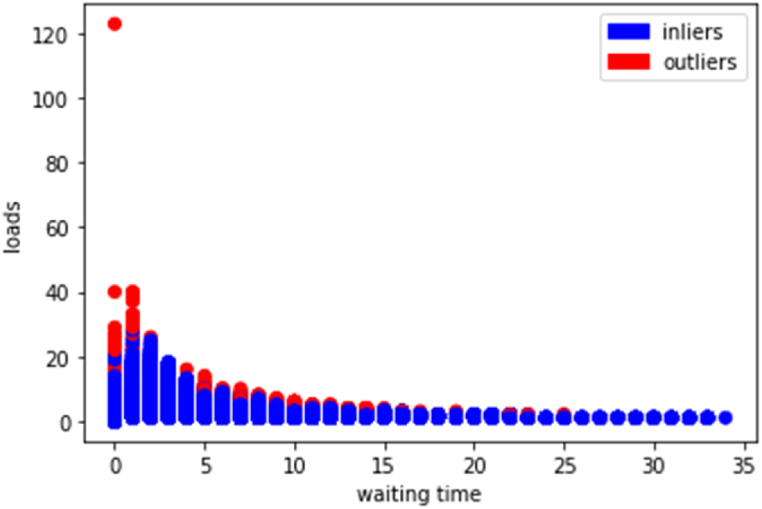
KNN results.

Once the distances are calculated, the K nearest neighbors are selected, and the algorithm determines whether the target data point x is an anomaly based on its proximity to these neighbors (see Fig. 6).

Table 4 summarizes the results obtained from each algorithm and cluster, providing a concise overview of the outcomes. This presentation facilitates a clearer understanding of the effectiveness of each algorithm in identifying anomalies within the dataset.

**Table 4:** Anomaly detection results.

Cluster	Number	HBOS	iForest	KNN
Group 0	15678	37	1	68
Group 1	16302	651	753	375
Group 2	43335	0	0	2

Sum	75315	688	754	445

A total of 1347 outliers were identified across all algorithms employed in the analysis. To facilitate a more comprehensive analysis, drivers detected by at least two algorithms were labeled accordingly. This labeling strategy ensures that anomalies identified by multiple algorithms receive heightened attention, potentially indicating more pronounced deviations from normal behavior. The share of each cluster is presented in Table 5.

**Table 5:** Anomaly detection in each cluster.

Anomaly detection	Group 0	Group 1	Group 2
485	8	477	0

In Group 1, anomalies account for 98 % of the loads detected, despite members spending significantly less time waiting in line. Conversely, the anomaly detection outcomes for Group 0 reveal that individuals in this group receive a load from the terminal only after acquiring a turn ticket, suggesting exceptionally long average waiting times (see Table 6).

**Table 6:** Anomaly detection in Group 0.

Number of turn ticket	Average of waiting time per load	Number of loads from terminal	Number of waybills
2	25	2	7
4	15	4	7
4	14	4	9
3	17	3	15
4	14	4	10
4	14	4	9
5	16	3	5
4	14	4	13

In the subsequent section, we will delve into the examination of an additional outlier from Group 1. While it is known that members of this group typically handle multiple loads and spend considerably less time waiting in line, upon comparing other members of this group with those identified through anomaly detection, several notable differences emerge (see Table 7).

**Table 7:** Group 1.

Group 1	Number of people	Number of turn tickets	Average of waiting time per load	Number of loads from the terminal
Other people	15825	10.2	2.9	7.96
Anomaly detection	477	20.5	1.9	19.6

Total	16302	10	3	8

As indicated in Table 7, the workload of these individuals is approximately 2.5 times higher than that of others, with their average wait time being less than one day. Examples of such individuals are illustrated in Table 7. Upon scrutinizing the waybills, it was observed that one particular individual acquired 123 loads from the terminal without accompanying waybills (see Table 8).

**Table 8:** Anomaly detection examples in Group 1.

Number of turn ticket	Total waiting time	Average waiting time	Total loads
124	0	0	123
26	0	0	26
26	0	0	26
28	0	0	28
25	0	0	25
23	0	0	23

## 5 Discussion

The careful selection of features played a crucial role in obtaining meaningful results in this study. For example, the inclusion of Ticket Count and Average Waiting Time allowed for the identification of drivers with disproportionately high workloads and minimal waiting times, highlighting anomalies that could be indicative of procedural inefficiencies or even fraudulent activities. Similarly, the Local feature provided valuable insights into geographical disparities, uncovering patterns that could be attributed to proximity advantages rather than operational inefficiencies. These findings emphasize the importance of targeted feature selection in anomaly detection, underscoring its potential to inform policy improvements and optimize operations within cargo terminals.

Outliers identified through the clustering and anomaly detection processes have significant economic implications for cargo terminal operations. Drivers displaying unusual waiting times or handling large numbers of loads within short time intervals may point to either operational inefficiencies or the possibility of fraudulent behavior. These anomalies, particularly in load handling and waiting time, do not only impact terminal throughput but may also highlight systemic issues or misconduct. This could potentially result in lost revenue, reduced productivity, and increased vulnerability to security breaches.

Our analysis revealed that the outliers primarily belonged to two distinct groups: drivers who handle high loads in brief periods with minimal waiting times (potential indicators of fraudulent behavior) and those who exhibit unusually long waiting times (potentially signaling inefficiencies or terminal bottlenecks). These patterns suggest a need for deeper investigation into terminal operations to mitigate the risks associated with both inefficiencies and criminal activities.

In this study, we began by clustering a dataset comprising 75,000 drivers into three distinct groups using the K-means algorithm: Group 0 drivers exhibit extended waiting times, Group 1 drivers handle loads rapidly, and Group 2 drivers exhibit typical behavior, serving as a reference group for comparison. Our results show that anomalies were predominantly concentrated within Group 0 and Group 1. To deepen our analysis, we applied point anomaly detection techniques using HBOS, Isolation Forest, and KNN algorithms. The results indicated that 98 % of the 485 anomaly detections were attributed to Group 1, with the remaining 2 % corresponding to Group 0.

Further analysis revealed that 477 members of Group 1 received loads approximately twice as frequently as their peers within the same group, despite spending considerably less time waiting in line. This observation points to anomalous load-receiving behavior among certain drivers, suggesting potential systemic issues in load allocation or even deliberate manipulation of the terminal's processes.

## 6 Future perspective

This paper delved into anomaly detection within terminals. For future research endeavors, we propose the utilization of machine learning techniques to predict load-receiving waiting times or to identify significant features associated with this aspect. Leveraging machine learning algorithms can offer enhanced predictive capabilities and enable the identification of key factors influencing waiting times, thereby contributing to more efficient terminal operations and improved overall performance.

During the preparation of this work the author(s) used ChatGPT in order to improve language and readability. After using this tool/service, the author(s) reviewed and edited the content as needed and take(s) full responsibility for the content of the publication.

## CRediT authorship contribution statement

**Shahab Emaani:** Writing – original draft, Formal analysis. **Abbas Saghaei:** Supervision.

## Declaration of competing interest

I am writing to declare that there are no financial or personal relationships with other individuals or organizations that could inappropriately influence the work presented in our manuscript entitled “Driver Anomaly Detection in Cargo Terminal” Therefore, we hereby confirm that there are no competing interests to disclose.
